# Association between vitamin D3 levels and insulin resistance: a large sample cross-sectional study

**DOI:** 10.1038/s41598-021-04109-7

**Published:** 2022-01-07

**Authors:** Zixin Xu, Rongpeng Gong, Gang Luo, Mingxiang Wang, Da Li, Yue Chen, Xiaofang Shen, Xiaoxing Wei, Niran Feng, Shuangquan Wang

**Affiliations:** 1 Shaanxi University of Chinese Medicine, Xi’an, 712046 Shaanxi People’s Republic of China; 2 Medical College of Qinghai University, Xining, 810016 Qinghai People’s Republic of China; 3 College of Eco-Environmental Engineering, Qinghai University, Xining, 810016 Qinghai People’s Republic of China; 4 Xi’an Chang’an District Hospital of Traditional Chinese Medicine, Xi’an, 710100 Shaanxi People’s Republic of China; 5 Department of Clinical Medicine, School of the First Clinical Medicine, Anhui Medical University, No. 81 Meishan Road, Hefei, 230032 Anhui People’s Republic of China; 6 Department of Nephrology, Aerospace Center Hospital, 15 Yuquan Road, Beijing, 100049 People’s Republic of China; 7 Tianjin University of Chinese Medicine, Tianjin, 301617 People’s Republic of China; 8 The Third Affiliated Hospital of Shaanxi University of Chinese Medicine, Xi’an XD Group Hospital, Xi’an, 710077 Shaanxi People’s Republic of China

**Keywords:** Diseases, Endocrinology

## Abstract

Previous studies have shown that vitamin D3 may be a potential factor in insulin resistance, but the relationship between vitamin D3 and insulin resistance still remains controversial. At present, more research is needed to explore the relationship between vitamin D3 and insulin resistance. The samples from 2009 to 2018 in NHANES database were analyzed to Investigate the relationship and the potential mechanism. We performed a cross-sectional study of five periods in the NHANES database. Finally, 9298 participants were selected through strict inclusion and exclusion criteria, Multivariate logistic regression analysis and curve fitting were conducted to explore the relationship between vitamin D3 level and insulin resistance. Moreover, subgroup analysis was used to further prove the association. The results revealed that there was a strong association between vitamin D3 and insulin resistance (OR 0.82, 95% CI 0.72–0.93). However, subgroup analyses indicated that this correlation varied between individuals and races. There was a negative correlation between vitamin D3 level and insulin resistance, which provides a new proof for exploring the influencing factors of insulin resistance. More well-designed studies are still needed to further elaborate on these associations.

## Introduction

Insulin resistance is a systemic metabolic disorder characterized by decreased insulin sensitivity^1^, which then progresses to a decrease in insulin action^2^. Insulin resistance is the basis of type 2 diabetes^1,3^ .Recently, the prevalence of diabetes has risen rapidly worldwide^4^, and type 2 diabetes is the most common type of diabetes, accounting for about 90% of all diabetic patients^5^. It was estimated that the number of patients with type 2 diabetes would reach up to 439 million by 2030^6^. Exploring the protective factors and risk factors of insulin resistance would help to take effective measures to prevent and reverse insulin resistance before type 2 diabetes^7^, and then to achieve the goal of controlling the incidence of type 2 diabetes.

Vitamin D3, which was also known as cholecalciferol, can be metabolized to produce 25-hydroxyvitamin D3^8^. Vitamin D3 can be supplemented through sunlight and food intake^9^. Vitamin D3 acted as a vital substance in human body, many diseases, such as type 2 diabetes^11^, cancer^12^, autoimmune diseases^13,14^, proteinuria^15^, hypertension^16^ would occur when vitamin D3 levels were below normal^10^. Previous studies have demonstrated that vitamin D3 might be a potential factor of insulin resistance^17^, while the research on the association between vitamin D3 and insulin resistance still remains controversial. Wallace et al. have conducted a double-blind, randomized, placebo-controlled experiment in 2019. The results revealed that vitamin D3 supplementation had no effect on insulin in pre-diabetes population^18^. However, a randomized, double-blind clinical trial with a sample size of 162 people in 2018 by Niroomand et al. showed that for patients with pre-diabetes and vitamin D3 deficiency, high-dose vitamin D3 supplementation can improve insulin sensitivity and reduce the risk of developing diabetes^19^. Considering the problems of small sample size and inconsistent results of the published articles, it is essential to perform an analysis to reassess the effect of vitamin D3 level on insulin resistance.

## Methods

### Database

All data in this study were obtained from the National Health and Nutrition Examination Survey (NHANES) database, which was a cross-sectional survey in the United States. The NHANES database contains demographic data, socio-economic data, dietary data and laboratory data and other data related to family health and nutrition. The Research Ethics Review Committee of the National Center for Health Statistics (NCHS) approved the NHANES project, and the data in NHANES were collected by professional investigators of NCHS. No application is required to use the database and it is available to any researcher who meets the requirements for use. All patient information in the database is anonymous, and all participants are aware of and consent to the data collection activities.

### Study population

**Figure 1:**
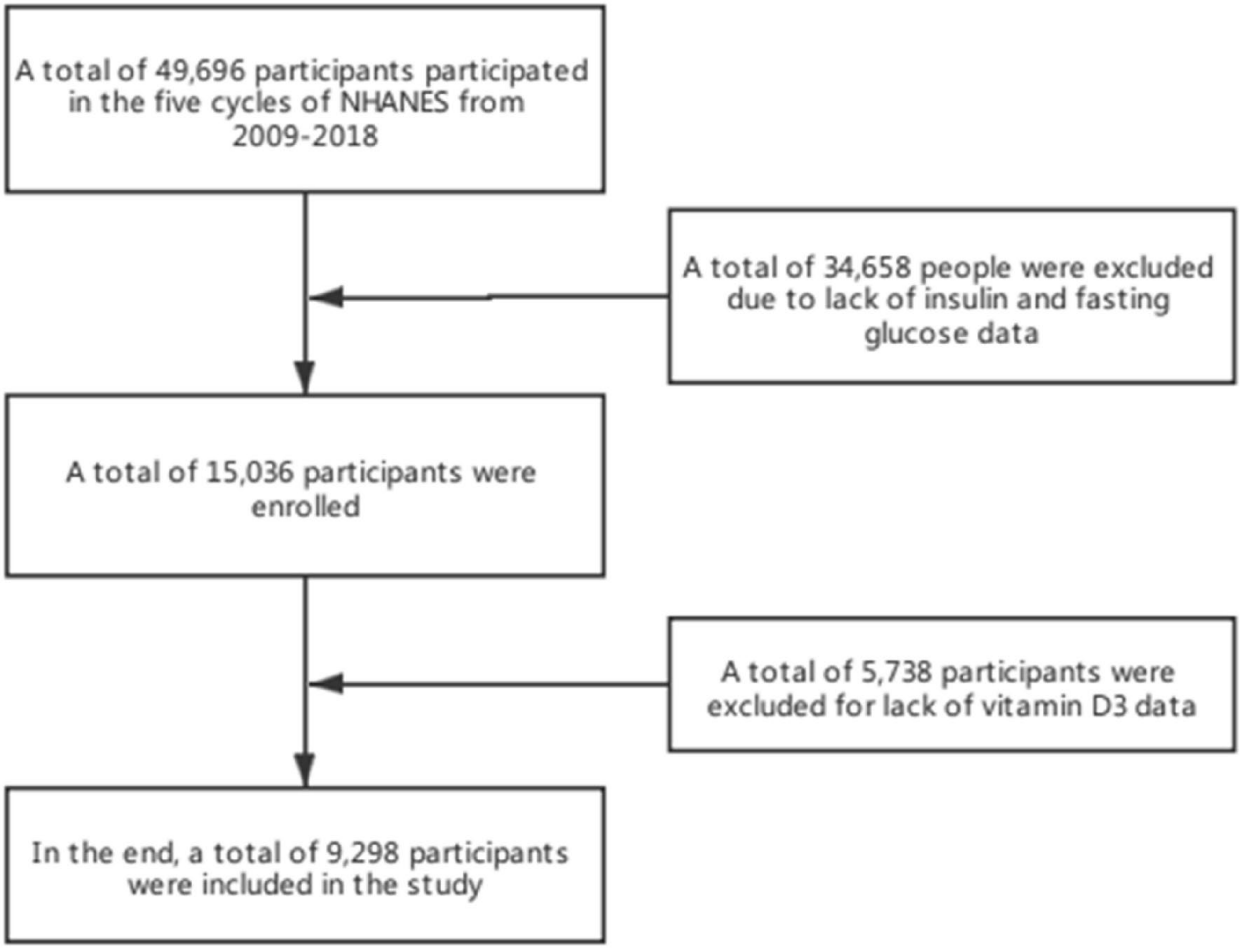
Flowchart of patient selection.

The data of NHANES database from 2009 to 2018 was selected. A total of 49,694 participants took the survey over the 10-year period. The inclusion and exclusion criteria were as follows (1) Determination of insulin and fasting blood glucose. (2) Determination of biochemical indexes such as vitamin D3, BUN and ALB (Fig. 1). Finally, a total of 9298 people were included in the study.

### Data collection

The data were obtained from the NHANES database on official website. We downloaded the demographic data, biochemical examination data, anthropometric data and health questionnaire data through the official website. These included sex, age, race, income, Vitamin D3, insulin, fasting blood glucose, glycosylated hemoglobin, albumin (ALB), Aspartate aminotransferase (AST), Blood urea nitrogen (BUN), height, weight, waist circumference, blood pressure, smoking, drinking and other data. Height and body mass index (BMI) was calculated from the height and weight data with the formula: BMI = weight (kg)/height squared (m^2^)^20^. Blood pressure data were measured several times and averaged.

### Statistical analysis

NHANES adopts complex and multi-stage probabilistic sampling design to select 5000 participants from different regions of American, which makes the data extensive and representative. Statistical analysis was carried out with R statistical software (R4.0.2), and the missing data was filled by multiple interpolation. In order to ensure the robustness of the analysis results, we performed sensitivity analyses on the interpolated data which showed that there was no obvious difference existed. Continuous variables are represented by detailed sample descriptions, and the average confidence interval is 95%. Classification variables are expressed in counts and weighted percentages. Normal distribution is described by median and standard deviation. Median and Q1–Q3 are used for skewness distribution.

We have developed four multivariate Logistic regression models. Model 1 was without adjusting any covariates; Model 2, adjusted for age, gender and race. Model 3 added smoking, drinking, income and education level as covariates as covariates to model 2; In model 4 added High-density lipoprotein (HDL), Urine creatinine (UCR), ALB, Waist, Alanine aminotransferase (ALT), AST, BUN, Total cholesterol (TC), γ-glutamyl transpeptidase (GGT), Lactate dehydrogenase (LDH), Total bilirubin (TBIL) and Uric acid (UA) to model 3. We also made a smooth fitting curve to explore the relationship between vitamin D3 level and insulin resistance. P value less than 0.05 (bilateral) is considered to have statistical significance. Construct subgroup analysis to further test the results.

### Covariant selection

In this study, based on the previous literature reports and clinical experience, we introduced the following covariates: age, sex, race, income, education, waist, UCR, ALB, ALT, AST, BUN, TC, GGT, LDH, TBIL, UA, smoking, drinking, blood pressure.

### Evaluation criterion

#### Insulin resistance

The measured fasting blood glucose value is multiplied by the insulin value and divided by 22.5, and the result value is recorded as HOMA-IR^21^, and those with HOMA-IR value greater than or equal to three-quarters digits are patients with insulin resistance^22^.

#### Hypertension

The average value of three blood pressure measurements was calculated, and the mean blood pressure was used to assess whether the participants are hypertensive. The diagnostic criteria of hypertension are systolic pressure ≥ 140 mmHg and/or diastolic pressure ≥ 90 mmHg^23^.

#### Drinking

We examined the classification of alcohol consumption in previous studies, which was finally divided into two levels. An alcoholic is defined as a person who drinks more than 12 drinks per year^24,25^.

#### Smoking

In this study, we divided smoking into two levels. Smoker refers to those who smokes more than 100 cigarettes in his whole life and still smokes^26^.

### Ethics approval and consent to participate

All participants provided written informed consent, and the study was approved by the NCHS Research Ethics Review Board (https://wwwn.cdc.gov/nchs/nhanes/default.aspx). The study was conducted in accordance with all relevant regulations and informed consent was obtained from all subjects and/or their legal guardians.

## Result

### Characteristics of study population

In this study, 49,696 participants were obtained from the NHANES database. After excluding 34,658 participants with insulin and fasting glucose deficiency data and 5738 participants with vitamin D3 deficiency data, a total of 2325 insulin-resistant patients and 6973 non-insulin-resistant patients were enrolled (Fig. 1). The average age of insulin-resistant patients (45.9 20.3) was slightly higher than those without insulin resistance (42.3 20.7). Among the people suffering from insulin resistance, 50.9% (1184 people) were male, 49.1% (1141 people) were female. 53% (1233) of insulin resistant people were drinkers, and 47% (1092) were non-drinkers. It was observed that BMI, WAIST, ALT, AST, GGT, LDH, UCR, UA levels of insulin resisters were higher than those of non-insulin resisters, while HDL, TBIL, ALB, VitD3 levels were lower than those of non-insulin resisters, but there was no significant difference in TC levels between the two groups (Table 1).

**Table 1:** Baseline characteristics of the study participants.

Variables	Total (n = 9298)	Non-insulin resistance (n = 6973)	Insulin resistance (n = 2325)	*P*-value
**Sex, n (%)**				0.022
Male	4542 (48.8)	3358 (48.2)	1184 (50.9)	
Female	4756 (51.2)	3615 (51.8)	1141 (49.1)	
Age, mean ± SD	43.2 ± 20.6	42.3 ± 20.7	45.9 ± 20.3	< 0.001
**Race, n (%)**				< 0.001
Mexican American	1511 (16.3)	1053 (15.1)	458 (19.7)	
Other Hispanic	956 (10.3)	713 (10.2)	243 (10.5)	
Non-Hispanic White	3824 (41.1)	2936 (42.1)	888 (38.2)	
Non-Hispanic Black	1876 (20.2)	1345 (19.3)	531 (22.8)	
Other Race—including Multi-Racial	1131 (12.2)	926 (13.3)	205 (8.8)	
**Education, n (%)**				< 0.001
Non-received higher education	4415 (47.5)	3181 (45.6)	1234 (53.1)	
Received higher education	4883 (52.5)	3792 (54.4)	1091 (46.9)	
**Income, n (%)**				< 0.001
Earning less than $1000,000	7836 (84.3)	5740 (82.3)	2096 (90.2)	
Earning more than or equal to $1000,000	1462 (15.7)	1233 (17.7)	229 (9.8)	
**Smoking, n (%)**				< 0.001
No	5952 (64.0)	4560 (65.4)	1392 (59.9)	
Yes	3346 (36.0)	2413 (34.6)	933 (40.1)	
**Alcohol use, n (%)**				0.026
NO	4180 (45.0)	3088 (44.3)	1092 (47)	
YES	5118 (55.0)	3885 (55.7)	1233 (53)	
BMI, mean ± SD	28.1 ± 7.1	26.2 ± 5.7	33.7 ± 7.8	< 0.001
Waist, mean ± SD	96.0 ± 17.5	91.2 ± 14.7	110.3 ± 17.5	< 0.001
HDL, median (IQR)	1.4 (1.2, 1.7)	1.4 (1.2, 1.8)	1.2 (1.0, 1.5)	< 0.001
TBIL, median (IQR)	12.0 (8.6, 15.4)	12.0 (10.3, 15.4)	10.3 (8.6, 13.7)	< 0.001
ALB, median (IQR)	43.0 (41.0, 45.0)	43.0 (41.0, 45.0)	42.0 (40.0, 44.0)	< 0.001
ALT, median (IQR)	20.0 (15.0, 27.0)	19.0 (15.0, 25.0)	24.0 (18.0, 34.0)	< 0.001
AST, median (IQR)	22.0 (19.0, 27.0)	22.0 (19.0, 26.0)	24.0 (20.0, 29.0)	< 0.001
BUN, median (IQR)	4.3 (3.2, 5.4)	4.3 (3.2, 5.4)	4.3 (3.2, 5.4)	0.004
TC, median (IQR)	4.7 (4.0, 5.5)	4.7 (4.0, 5.4)	4.7 (4.1, 5.5)	0.243
GGT, median (IQR)	18.0 (13.0, 27.0)	16.0 (12.0, 24.0)	23.0 (16.0, 37.0)	< 0.001
Vit D3, median (IQR)	57.2 (41.0, 74.5)	59.2 (42.9, 76.4)	51.0 (36.0, 67.8)	< 0.001
LDH, median (IQR)	125.0 (111.0, 143.0)	124.0 (110.0, 142.0)	129.0 (114.0, 147.0)	< 0.001
UCR, median (IQR)	180.0 (92.0, 6630.0)	173.0 (86.0, 6100.0)	205.0 (106.0, 8663.0)	< 0.001
UA, median (IQR)	315.2 (261.7, 374.7)	303.3 (255.8, 362.8)	350.9 (291.5, 410.4)	< 0.001

*BMI* body mass index, *HDL* high density lipoproteinm, *TBIL* total bilirubin, *ALB* albumin, *ALT* alanine aminotransferase, *AST* aspartate aminotransferase, *BUN* blood urea nitrogen, *TC* total cholesterol, *GGT* γ-glutamyl transpeptidase, *Vit D3* vitamin D3, *LDH* lactate dehydrogenase, *UCR* urine creatinine, *UA* uric acid.

### Single factor analysis

Univariate logistic regression was performed to analyze the relationship between the occurrence of insulin resistance and hypercholesterolemia by gender, age, race, education level, income level, hypertension, VitD3, WAIST, ALT, AST, HDL and BUN. We found that women were less likely to develop insulin resistance than men, with an OR of 0.9 (95% CI 0.81–0.98). The probability of insulin resistance in people with higher education level is lower than that with lower education level, and the confidence interval between OR value and 95% CI is 0.74 (0.68, 0.81). Smokers, non-drinkers, people with hypertension and hypercholesterolemia are more likely to develop insulin resistance. The levels of LDH and VitD3 were negatively correlated with the occurrence of insulin resistance, while the values of ALT, AST, GGT, BUN and UA were positively correlated with the occurrence of insulin resistance (Table 2).

**Table 2:** Univariate analysis for insulin resistance.

Variables	OR (95% CI)	*P*-value
**Gender**		
Male	1	
Female	0.9 (0.81–0.98)	0.021
Age	1.01 (1.01–1.01)	< 0.001
**Race, n (%)**		
Mexican American	1	
Other Hispanic	0.78 (0.65–0.94)	0.009
Non-Hispanic White	0.7 (0.61–0.79)	< 0.001
Non-Hispanic Black	0.91 (0.78–1.05)	0.202
Other race—including multi-racial	0.51 (0.42–0.61)	< 0.001
**Education, n (%)**		
Non-received higher education	1	
Received higher education	0.74 (0.68–0.81)	< 0.001
**Income, n (%)**		
Earning less than $1000,000	1	
Earning more than or equal to $1000,000	0.51 (0.44–0.59)	< 0.001
Waist	1.08 (1.07–1.08)	< 0.001
**Alcohol use**		
No	1	
Yes	0.9 (0.82–0.99)	0.024
**Smoking, n (%)**		
No	1	
Yes	1.27 (1.15–1.39)	< 0.001
HDL	0.22 (0.19–0.25)	< 0.001
ALT	1.02 (1.02–1.03)	< 0.001
AST	1.01 (1–1.01)	< 0.001
GGT	1.01 (1.01–1.01)	< 0.001
BUN	1.04 (1.02–1.07)	< 0.001
UA	1.01 (1.01–1.01)	< 0.001
Vit D3 log	0.52 (0.47–0.57)	< 0.001
**Hypertension**		
No	1	
Yes	2.31 (2.1–2.54)	< 0.001
**Hypercholesterolemia**		
No	1	
Yes	1.6 (1.46–1.76)	< 0.001

*BMI* body mass index, *HDL* high density lipoprotein, *TBIL* total bilirubin, *ALB* albumin, *ALT* alanine aminotransferase, *AST* aspartate aminotransferase, *BUN* blood urea nitrogen, *TC* total cholesterol, *GGT* γ-glutamyl transpeptidase, *Vit D3* vitamin D3, *LDH* lactate dehydrogenase, *UCR* urine creatinine, *UA* uric acid.

### Multivariate analysis

In this study, we constructed four models to analyze the relationship between VitD3 and insulin resistance. Model 1 was without adjusting any covariates; Model 2, adjusted for age, gender and race. Model 3 added smoking, drinking, income and education level as covariates as covariates to model 2; In model 4 added HDL, UCR, ALB, waist, ALT, AST, BUN, TC, GGT, LDH, TBIL and UA to model 3. The effect value based on the model can be interpreted as a corresponding percentage reduction in the risk of insulin resistance when vitamin D3 is increased by one unit. For example, in the unadjusted model, the OR value is 0.52 (0.47, 0.57), which means that the risk of insulin resistance is reduced by 48% for every additional unit of vitamin D3. In the model (Model 2) adjusted only for the socio-demographic data, the risk of insulin resistance decreased by 52% [0.48 (0.43, 0.54)] for each additional unit of VitD3. In the further adjusted model (Model 3), the risk of insulin resistance is reduced by 50% [0.50 (0.45, 0.56)] for each additional unit of VitD3. In the fully adjusted model (model 4), the risk of insulin resistance decreased by 18% [0.82 (0.72, 0.93)] for each additional unit of VitD3. For sensitivity analysis, we transform VitD3 from continuous variable to classified variable. The p value of VitD3 trend in model 4 is consistent with the result when VitD3 is a continuous variable. In addition, we also found that the effects of different VitD3 groups showed an equidistant trend (Table 3).

**Table 3:** The association between Vit D3 and insulin resistance in a multiple regression model.

Outcome	Model 1		Model 2		Model 3		Model 4	
	OR (95% CI)	*P*-value	OR (95% CI)	*P*-value	OR (95% CI)	*P*-value	OR (95% CI)	*P*-value
vitamind3	0.52 (0.47–0.57)	< 0.001	0.48 (0.43–0.54)	< 0.001	0.50 (0.45–0.56)	< 0.001	0.82 (0.72–0.93)	0.003

Model 1: No adjustments have been made; Model 2: Adjustments were made for gender, age and race; Model 3: Adjusted for model 2, smoking, alcohol consumption, income, and education level were added; Model: HDL, UCR, ALB, WAIST, ALT, AST, BUN, TC, GGT, UA, TBIL, LDH were added on the basis of model 3.

### Smooth curve fitting

**Figure 2:**
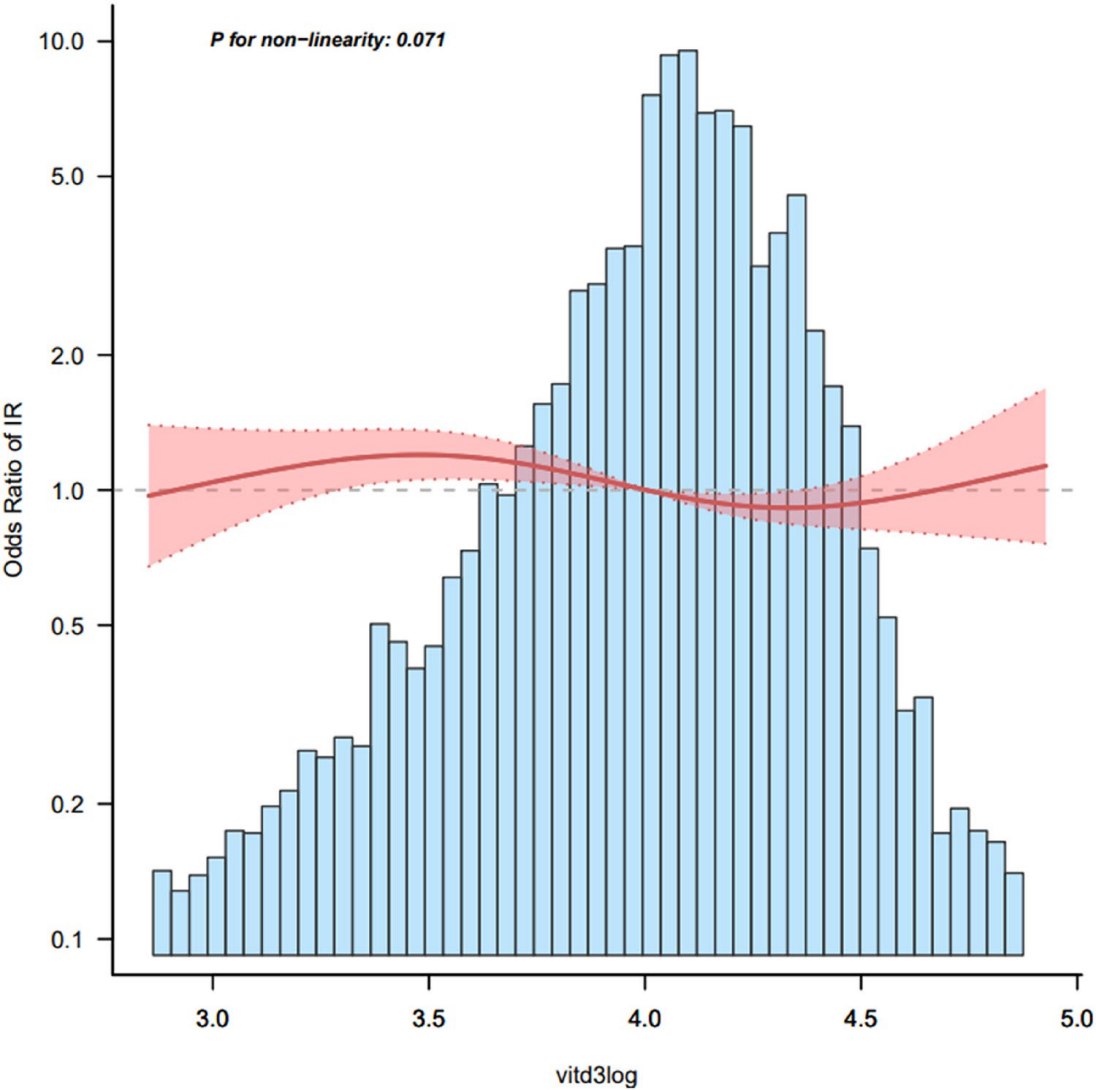
Association between Vitamin D3 and insulin resistance.

Besides, we analyzed the linear relationship between vitamin D3 and the occurrence of insulin resistance (Fig. 2). The results of smooth curve and logistic regression showed that after adjusting for gender, age, race, education level, income, HDL, UCR, ALB, waist circumference, ALT, AST, BUN, TC, GGT, smoking, drinking, LDH, TBIL, UA, there was a linear relationship between vitamin D3 and insulin resistance (P for non-linearity = 0.01) There is a negative correlation between vitamin D3 level and insulin resistance (Fig. 2).

### Subgroup analysis

**Figure 3:**
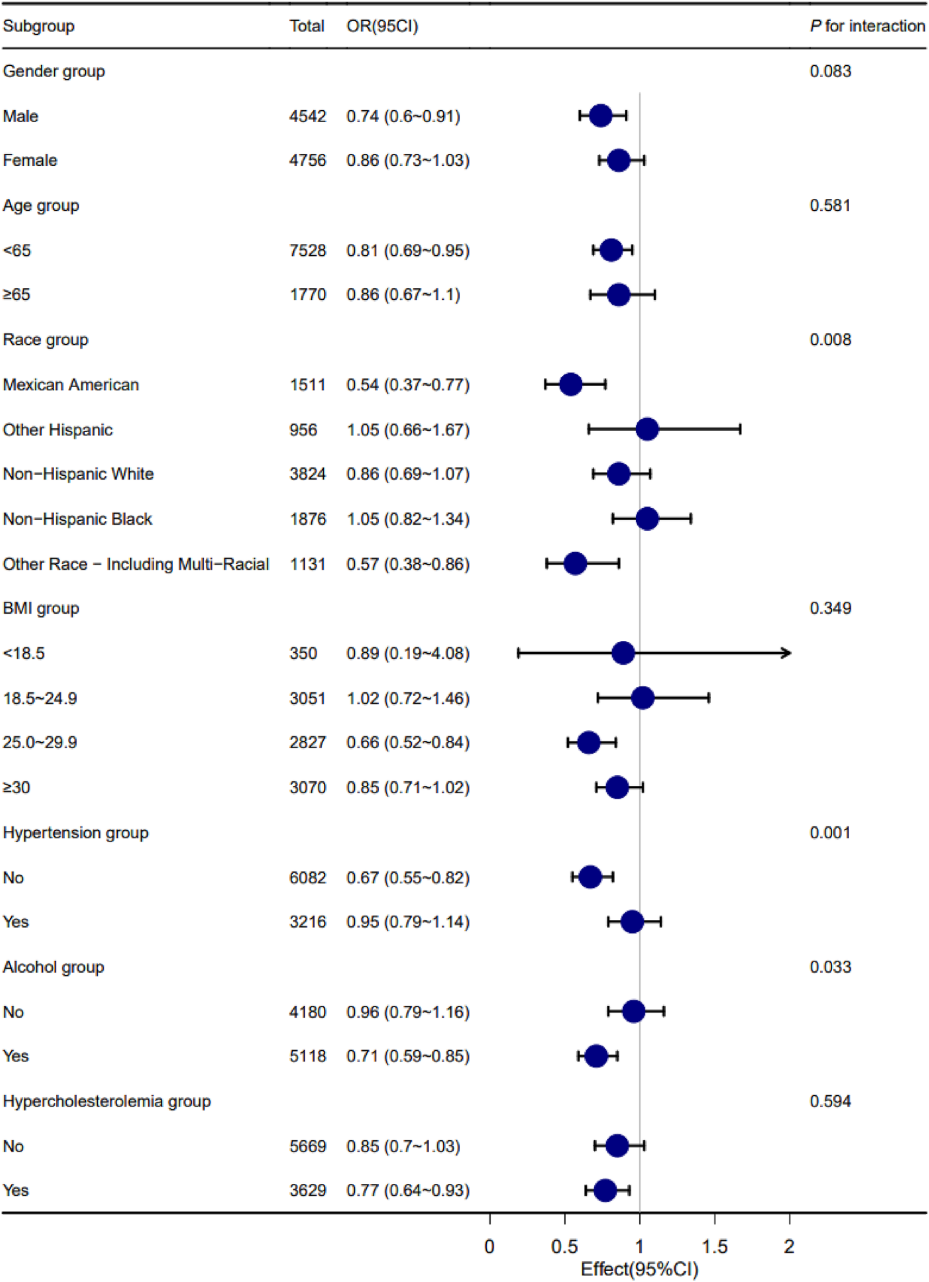
Subgroup analysis.

In order to better explain this result, we conducted subgroup analysis and interaction test. According to whether the age is less than 65 years old or not, it can be divided into two parts: less than 65 years old and greater than or equal to 65 years old^27,28^. According to WHO standards, BMI is divided into four parts: < 18.5 kg/m^2^, 18.5 ~ 24.9 kg/m^2^, 25.0 ~ 29.9 kg/m^2^ and ≥ 30.0 kg/m^2^^29,30^. According to age, sex, race, BMI, hypertension and hypercholesterolemia, this study verified whether the relationship between VitD3 level and insulin resistance was still applicable in each subgroup. The results showed that there was no obvious interaction between VitD3 level and insulin resistance in hypercholesterolemic and non-hypercholesterolemic people and in different age, BMI and gender groups. There is an interaction between VitD3 level and insulin resistance in hypertensive and non-hypertensive people, drinking and non-drinking people and different ethnic groups. We found that vitamin D3 has a stronger correlation with insulin resistance in non-hypertensive people than in hypertensive people. Compared with non-drinkers, the correlation between vitamin D3 and insulin resistance in drinkers is stronger. This suggests that we should maintain a higher level of vitamin D3 in hypertension and non-drinkers in order to prevent insulin resistance. Vitamin D3 is a protective factor of insulin resistance in Mexican–American, Non-Hispanic-White and other race-including multi-racial, and its effect value and 95% confidence interval are 0.54 (0.37–0.77), 0.86 (0.69–1.07) and 0.57 (0.38–0.86), respectively Vitamin D3 is a risk factor for insulin resistance in Other-Hispanic and Non-Hispanic-Black. (Fig. 3).

## Discussion

Cross-sectional study is considered as a critical tool to assess the effects of treatment or risk factors for disease. Therefore, we conducted this research which identified a 0.82-fold decrease in the risk of developing insulin resistance in patients who take vitamin D3 for each additional unit of vitamin intake (OR 0.82, 95% CI 0.72–0.93) in fully adjusted model (model 4). This might indicate that vitamin D3 is a protective factor in the occurrence of insulin resistance. This may be because vitamin D3 can effectively inhibit the occurrence of inflammation^31,32^, and inflammation is the main factor inducing insulin resistance. Vitamin D3 can inhibit the occurrence of inflammatory reaction by up-regulating MAP kinase, regulating NF-kB signaling pathway, regulating cytokine level and prostaglandin pathway^33,34^, and then achieve the purpose of reducing insulin resistance.

Univariate Logistic analysis showed that the incidence of insulin resistance in drinkers was lower than that in non-drinkers. The results of subgroup analysis also confirm this point. Studies have shown that moderate drinking can reduce the risk of insulin resistance^35,36^. Studies have shown that glutathione is involved in the synthesis of liver insulin sensitizers, and drinking alcohol can increase the level of glutathione in liver, so drinking alcohol can improve insulin sensitivity and reduce the risk of insulin resistance^37,38^. Studies have shown that there is a correlation between proper drinking and lower risk of type 2 diabetes^39^, which is consistent with our results. It is important to emphasize that excessive alcohol consumption can lead to obesity, which is closely related to insulin resistance^40,41^. And alcohol itself is also easy to cause a variety of diseases, and everyone's tolerance to alcohol is different. Therefore, it is difficult to set a standard of alcohol consumption that is suitable for most people to prevent insulin resistance without affecting other health indicators. It is important to note that moderate alcohol consumption is not the best way to prevent insulin resistance, although studies have shown that moderate alcohol consumption is beneficial to prevent insulin resistance. Notably, one RCT study showed that caloric restriction was beneficial for weight loss on both high- and low-glycemic diets. A healthy diet seems to be a more appropriate way to prevent insulin resistance^42^.

Subgroup analysis showed that the relationship between vitamin D3 and insulin resistance was different among different races (P = 0.008). Vitamin D3 is the protective factor of insulin resistance in Mexican–American, Non-Hispanic-White and other race-including multi-racial, while vitamin D3 is the risk factor of insulin resistance in Other-Hispanic and Non-Hispanic-Black (Fig. 3). This shows that the relationship between vitamin D3 level and insulin resistance is different among different races. Previous studies have shown that the insulin sensitivity of different ethnic groups is different^43^. Studies have shown that the way of synthesizing D3 by ultraviolet rays in dark-skinned individuals is inhibited^44,45^. This may be due to excessive melanin blocking ultraviolet rays and affecting the production of vitamin D3. At the same time, different races have different levels of vitamin D3, and the content of vitamin D3 in blacks is obviously lower than that in whites^46^. At the same time, the relationship between vitamin D3 and insulin resistance is also different between hypertensive patients and non-hypertensive people (P = 0.001). We found that vitamin D3 has a stronger correlation with insulin resistance in the non-hypertensive population than in the hypertensive population (Fig. 3). This may indicate that hypertension can weaken the relationship between vitamin D3 and insulin resistance. Previous studies have shown that hypertension can cause insulin resistance by changing the way of delivering insulin and glucose to skeletal muscle cells^47^ and vitamin D3 deficiency can increase the risk of hypertension^16,48^. We speculate that the decrease of vitamin D3 level will increase the risk of hypertension and insulin resistance, and the occurrence of hypertension will further promote the occurrence of insulin resistance. This suggests that people with hypertension need to maintain a higher level of vitamin D3 to prevent insulin resistance. This conclusion needs more experimental and clinical studies to verify and clarify its mechanism.

In previous studies, we found a slightly weaker correlation between vitamin D3 levels and diabetes in the hyperuricemic population than in the non-hyperuricemic population. This proves that vitamin D3 plays a certain role in the pathogenesis of diabetes, and this role also applies to hyperuricemia. In this study, we found that vitamin D3 is related to insulin resistance, which further confirmed the role of vitamin D3 in the pathogenesis of diabetes^4^.

Currently, studies have found a linear relationship between vitamin D3 and insulin resistance. Therefore, within a certain range, maintaining higher vitamin D3 level is essential to prevent insulin resistance, and this result is a guideline for clinical practice. However, more cohort studies are needed to validate this conclusion.

Several limitations of this study should be mentioned. (1) The time span of this study is long and there are differences in the way insulin resistance is judged. However, our current method allows for a much larger number of samples to be included, and the detection method is very clear. Considering that all samples are detected by professional testing facilities, It is considered that this effect can be ignored. (2) It is acknowledged that there are some bias inevitably existed in cross sectional study. We will conducted the cohort study in the future when conditions permit. (3) The population observed in this study is American, and special populations such as pregnant women and cancer patients are not included. Due to the limited sample, we cannot analyze the special population and more other races. Therefore, whether this result is applicable to special people and people from other countries needs further study. We will collect such samples for analysis in future studies to make up for the shortcomings of this study.

Despite these limitations, our research had some notable advantages. (1) The amount of data used in this study is large and generalized. (2) The NHANES database is an internationally recognized high-quality database with comprehensive and reliable data that greatly enriches research data. (3) In this study, a more advanced multiple interpolation method was applied to deal with the missing data, and the sensitivity of the interpolated data was analyzed. The results show that there is little difference between the interpolated data and the original data, which makes our results more convincing.

## Conclusion

In conclusion, the results of this cross-sectional study based on the data of five cycles (2009–2018) in American NHANE database demonstrated that there is a correlation between vitamin D3 level and insulin resistance. However, more studies are urgely needed to investigate whether this conclusion is still applicable to special populations. This study provides a new way to explore the influencing factors of insulin resistance. In the future, more factors related to the occurrence of insulin resistance would be found in different populations, which would provide more accurate and effective prevention and treatment programs to prevent insulin resistance and type 2 diabetes.

## Abbreviations

BMI: Body mass index
FBG: Fasting blood-glucose
VitD3: Vitamin D3
Hb-A1c: Glycosylated hemoglobin;
TC: Total cholesterol
ALT: Alanine aminotransferase
BUN: Blood urea nitrogen
W.C: Waist
TG: Triglyceride
CI: Confidence interval
OR: Odds ratio
HUA: Hyperuricemia
